# Short-Term Impacts of Meteorology, Air Pollution, and Internet Search Data on Viral Diarrhea Infection among Children in Jilin Province, China

**DOI:** 10.3390/ijerph182111615

**Published:** 2021-11-04

**Authors:** Wengao Lu, Jingxin Li, Jinsong Li, Danni Ai, Hong Song, Zhaojun Duan, Jian Yang

**Affiliations:** 1 Department of Computer Science and Technology, Beijing Institute of Technology, Beijing 100081, China; luwengao2007@163.com (W.L.); songhong@bit.edu.cn (H.S.); 2 National Institute for Viral Disease Control and Prevention, Chinese Center for Disease Control and Prevention, Beijing 102206, China; costaroy@163.com (J.L.); zhaojund@126.com (Z.D.); 3 Laboratory of Beijing Engineering Research Center of Mixed Reality and Advanced Display, School of Optics and Photonics, Beijing Institute of Technology, Beijing 100081, China; jyang@bit.edu.cn

**Keywords:** viral diarrhea, mean temperature, precipitation, air quality index, combined Baidu search index, DLNM

## Abstract

The influence of natural environmental factors and social factors on children’s viral diarrhea remains inconclusive. This study aimed to evaluate the short-term effects of temperature, precipitation, air quality, and social attention on children’s viral diarrhea in temperate regions of China by using the distribution lag nonlinear model (DLNM). We found that low temperature affected the increase in children’s viral diarrhea infection for about 1 week, while high temperature and heavy precipitation affected the increase in children’s viral diarrhea infection risk for at least 3 weeks. As the increase of the air pollution index may change the daily life of the public, the infection of children’s viral diarrhea can be restrained within 10 days, but the risk of infection will increase after 2 weeks. The extreme network search may reflect the local outbreak of viral diarrhea, which will significantly improve the infection risk. The above factors can help the departments of epidemic prevention and control create early warnings of high-risk outbreaks in time and assist the public to deal with the outbreak of children’s viral diarrhea.

## 1. Introduction

Acute gastroenteritis is a common disease of the digestive system, which is characterized by symptoms including vomiting, diarrhea, and fever. Many external factors lead to acute gastroenteritis, including bacteria, viruses, and parasites. Viral diarrhea is a common digestive system disease caused by various human enteroviruses, including rotavirus, norovirus, adenovirus, and astrovirus [1]. The main susceptible population is children under 5 years old [2]. Viruses are considered the main pathogen of severe acute diarrhea among children worldwide, and they are also one of the main causes of children’s death in developing countries [3].

Relevant research shows that norovirus is a typical foodborne virus [4], which is easily transmitted through unclean water sources and unclean foods. Rotavirus can form aerosols with pollutants in the air and spread through fecal-oral cavities or contact with pollutants [5]. As the transmission route of viral diarrhea is directly close to humans’ daily lives, viral diarrhea can spread extensively all over the world and break out all year round.

The regularity of viral diarrhea is ascribed to various environmental factors, including various meteorological and hydrological factors. In the available evidence, low temperatures and drought facilitate the spread of rotavirus, and rotavirus associated with diarrhea exhibits seasonal characteristics in temperate regions. However, the epidemic pattern of norovirus is irregular, and its peak may shift within weeks or months, showing high seasonal variability [6]. A previous study found that children born in summer are at higher risk of rotavirus infection in England and Wales than those born in other seasons [7]. Studies in Britain, Netherlands [8], Turkey [9], Australia [10], Germany [11], India [12], Costa Rica [13], Nepal [14], and other parts of the world show that the risk of diarrhea caused by rotavirus is negatively correlated with temperature. In some other areas, such as Bangladesh [15], the risk of the rotavirus outbreak increases due to high temperatures. The increase in runoff [16] and water level [15] of rivers can promote the outbreak of diarrhea from the norovirus. Research on seafood farming environments shows that factors including solar radiation, water temperature, and salinity can affect the virus-carrying capacity of marine products as the host of norovirus, thereby further affecting the outbreak of foodborne norovirus in public [17].

In China, the association analysis of environmental factors on acute gastroenteritis and bacterial diarrhea has been widely reported, but reports of environmental factors on viral diarrhea are limited. Wang, P. studied the seasonal variation in the number of hospitalizations infected by norovirus and rotavirus in Hong Kong, China, and found that rotavirus is likely to break out in winter, while norovirus is associated with summer [18]. Compared with micro rainfall, the risk of norovirus infection is higher but the risk of rotavirus infection is lower under extreme rainfall. Gao, Y. investigated environmental temperature and viral diarrhea infection burden in Wuxi, China, and found that low temperatures can promote the outbreak of viral diarrhea, which is consistent with research in other parts of the world [19]. Ye, Q. investigated the relationship between air pollutants and the rotavirus infection rate on children in Hangzhou, China [20]. They further verified the negative correlation between temperature and the rotavirus infection rate and found that the temperature change has a significant impact on the rotavirus detection rate. Notably, they found that the increase in the PM2.5 concentration, PM10 concentration, and the concentration of other air pollutants can significantly increase the risk of rotavirus infection; they observed dose, lag, and cumulative effects.

To date, researchers have pointed out the significance of tracking and monitoring infectious diseases by using Internet search data. For example, in the United States, Google search data can report influenza trends 2 weeks in advance [21]. Other researchers also use search query data to detect the incidence of dengue fever [22], Ebola virus [23], hand-foot-mouth disease [24], and other infectious diseases. Based on the composite Baidu index and norovirus incidence data with different time delays, Liu, K. built an exponential curve model with the Spearman correlation method to fit the norovirus epidemic in Zhejiang Province, China, in 2014 and found that the risk of norovirus infection increased by 2.15 times for each additional unit of the average composite Baidu index [25]. Given that Internet monitoring data comes from social media, search engine query data, and news, using Internet search data can improve the sensitivity and timeliness of detecting health events [26]. However, external interference such as media, Internet use behavior, and regional policies may bring many deviations and influence the accuracy of health event predictions. Therefore, using Internet search data alone to monitor the occurrence of infectious diseases has certain limitations [27]. We speculate that the combination of Internet query data and traditional monitoring may improve the accuracy of infectious disease monitoring and make appropriate predictions and early warnings for the outbreak of infectious diseases.

This study aimed to explore the lag dependence of meteorological factors, air quality factors, and Internet search data on the risk of viral diarrhea among children under 5 years old in temperate regions of China. We aimed to provide perspectives of external natural environmental factors and social activity factors for the risk of viral diarrhea among children and assist local health departments to better prevent and control the outbreak of viral diarrhea.

## 2. Materials and Methods

### 2.1. Research Area

Jilin Province, located in northeast China, has a temperate monsoon climate with short, hot, humid summers and cold and dry winters, which last for nearly half a year. Jilin Province has eight prefecture-level cities and one autonomous prefecture, with a population of about 27 million and an urbanization rate close to 60%. In 2018, the per capita GDP was slightly higher than 8000 US dollars, which led to a middle-income region. According to the analysis report of the service industry of China Mobile Internet in 2018, the Internet penetration rate in Jilin Province exceeded 50% in 2016 [28].

### 2.2. Data Collection

Viral diarrhea is a common public health problem in China, and it is listed as a Class C notifiable infectious disease. In 2003, the Chinese government constructed a national notifiable infectious disease reporting system, which requires clinicians to report the personal information of patients online to China’s CDC using a standardized form within 24 h after patients are diagnosed. In this study, the data of viral diarrhea cases among children under 5 years old in Jilin Province from 2014 to 2019 were collected from the National Institute for Viral Disease Control and Prevention of China. Each case contained personal information, including gender, age, infection date, and the category of pathogenic virus.

The meteorological data were obtained from the China Meteorological Data Service Center (http://data.cma.cn, accessed on 11 December 2020), which is a component of the National Science and Technology Infrastructure Platform of China Meteorological Administration. It provides a variety of meteorological time series data, which can be characterized by specific numerical values. The daily average temperature (°C) and daily precipitation (mm) were selected to build the nonlinear model. In our study, the daily average temperature and precipitation recorded by 30 monitoring points in Jilin Province were arithmetically averaged by day, and the daily average temperature and daily precipitation of Jilin Province were obtained. The air quality index (AQI) data were obtained from the air quality online monitoring and analysis platform of China (https://www.aqistudy.cn/, accessed on 11 December 2020). From this platform, the daily AQI of nine municipal administrative regions of Jilin Province was obtained, and the values were arithmetically averaged by day to form the daily AQI of Jilin Province. In China, Baidu is the search engine with the highest market share. The National Institute for Viral Disease Control and Prevention of China provided up to 20 associated keywords (listed in Table S1 in Supporting Information) according to the symptoms, pathogenic factors, and medical products for the prevention and treatment of viral diarrhea. We obtained the data of the Baidu search index of the 20 keywords in Jilin Province in the corresponding period for this study.

### 2.3. Statistical Analysis

The epidemiological data of viral diarrhea among children under 5 years old in Jilin Province were descriptively analyzed. We statistically analyzed the average, standard deviation, and time series of daily children’s viral diarrhea cases and various selected external factors. The Pearson correlation test was used to evaluate the relationship between the number of daily viral diarrhea infections and external factors. The correlation and significance between epidemiological data and the external factors were obtained. The external factors whose absolute value of correlation coefficient with the epidemiological data exceeded 0.1 and with statistical significance (*p* < 0.05) were selected for further analysis.

Given that we obtained up to 20 columns of the Baidu search index series, to simplify the research process, we designed a combined Baidu index to describe the comprehensive search data, as shown in Equation (1).

(1) $$CBDI=\sum_{i=1}^{n}\beta_{i}x_{i},$$

In Equation (1), *CBDI* is the combined Baidu index. *x_i_* and *β_i_* represent the data of each selected Baidu search index column and the Pearson correlation coefficient between each selected column and the epidemiological data, respectively. *n* is the number of selected Baidu search index columns.

The DLNM is a regression model based on the lag effect [29], which can analyze the lag effect and cumulative effect of single or hybrid elements in a nonlinear process. In this study, we brought the daily average temperature, precipitation, *AQI*, and the combined Baidu index into the nonlinear model, as described in Equation (2). The “dlnm” package in R software was used to build the nonlinear model for further data analysis [30].

(2) $$\begin{array}{r} Log(E[Y_{t}])=cb(MT,df_{MT};lag=21,df_{lag})+cb(P,df_{P};lag=21,df_{lag})\\ +cb(AQI,df_{AQI};lag=21,df_{lag})+cb(CBDI,df_{AQI};lag=21,df_{CBDI})\\ +ns(date,df=7/year)+DOW_{t}+Season_{t} \end{array}$$

In Equation (2), *E*[*Y_t_*] is the time series of daily viral diarrhea infections among children under 5 years old; *cb*(*MT*), *cb*(*P*), *cb*(*AQI*), and *cb*(*CBDI*) are the cross-basis matrix of time series of daily average temperature, daily precipitation, daily AQI, and daily combined Baidu index, respectively. Natural cubic spline function was adopted in all the element spaces, where spline nodes were selected from 25%, 50%, and 75% quantiles of the logarithmic scale of each external factor, and the initial degree of freedom (*df*) was 3. According to previous research experience on the exposure risk of environmental factors to bacterial diarrhea, a lag period of 21 days was selected when establishing a cross-basis matrix for various factors in our study, and the corresponding initial *df* was 3. The mixed elements of the nonlinear model included time, day of the week, and season. The time factor adopting a natural cubic spline function was used to control the long-term trend, and the corresponding initial value of *df* was set as 7 per year. The quasi-Poisson function was used as the connection function in the model to control the over-dispersion effect. Through the above model, the relative risk of viral diarrhea infection among children on a certain day and the cumulative risk of external factors to viral diarrhea infection under different values could be obtained. Relative risk refers to the ratio of the probability of infection in the exposed group to that in the non-exposed group. In the nonlinear model, the reference values of the four external elements were set as the average values of the corresponding data columns. None of any interventions were conducted during the period.

The sensitivity of the nonlinear model was analyzed by changing the *df* values of each cross-basis matrix of different external factors, deleting seasonal factors. Akaike Information Criterion was used to evaluate the model and determine the final values of *df*. The model with the minimum AIC was selected as the optimal one. We also conducted subgroup analyses. The viral diarrhea cases among children under 5 years old were divided into subgroups according to gender (male and female) and age (0–1, 1–2, and 2–5 years old), respectively. The nonlinear model illustrated in Equation (2) was applied to these subgroups for further study.

## 3. Results

### 3.1. Epidemiology and Descriptive Statistics Analysis

**Figure 1:**
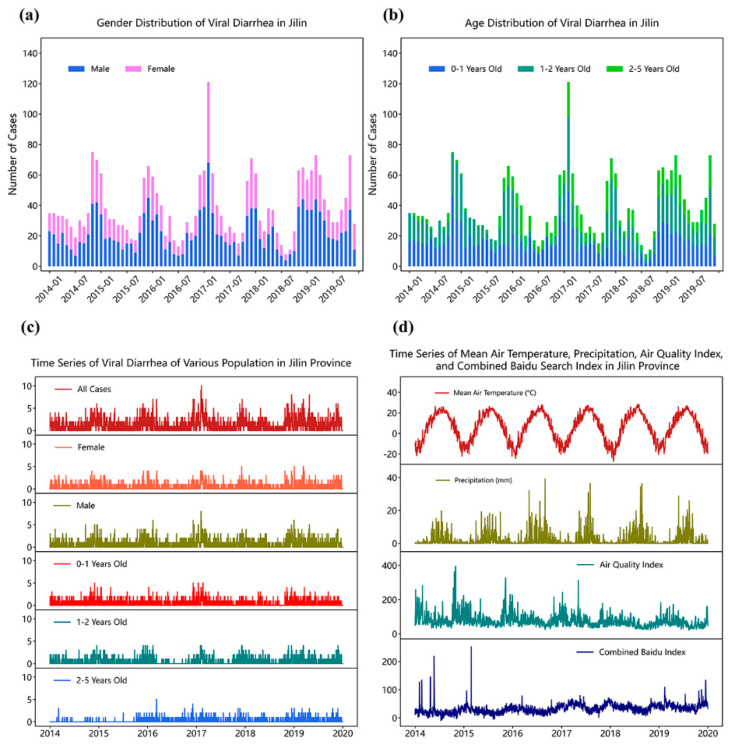
Epidemiology of viral diarrhea among children under 5 years old in Jilin Province from 2014 to 2019 and time-series data of the selected external factors. (**a**) Gender distribution of monthly cases of viral diarrhea; (**b**) Age distribution of monthly cases of viral diarrhea; (**c**) Time series of the total number of viral diarrhea cases among children and those in different subgroups; (**d**) Time series of the selected external factors.

From 2014 to 2019, 2794 cases of viral diarrhea were recorded among children under 5 years old in Jilin Province, and the monthly epidemic pattern is shown in Figure 1. Viral diarrhea among children under 5 years old in Jilin Province exhibited obvious seasonality, with a high incidence in winter every year. The number of cases in December accounted for 12.88% of the total cases, which was the highest, followed by 12.81% in November, 12.03% in January, and 11.99% in February. The proportion of cases in summer and autumn was relatively low, and the proportion of cases from June to September was about 5%. Figure 1a shows the gender distribution of viral diarrhea among children, in which males accounted for 58.3% while females accounted for 41.7%, and the ratio of males to females was 1.4:1. Figure 1b shows the distribution of age groups, and the proportion of cases of infants under 1 year old accounted for 41.84%, which was significantly higher than that of other age groups. Children aged from 1 to 2 years old accounted for 38.33%, and children aged from 2 to 5 years old accounted for less than one-fifth. In this study, we analyzed all viral diarrhea cases of children under 5 years old and the subgroups of children of different gender and age groups. Table 1 summarizes the descriptive statistics of the daily number of infection cases of each group, daily average temperature, precipitation, air quality index, and composite Baidu search index from 2014 to 2019. The time series of daily infection numbers in each group and four selected external factors are shown in Figure 1c,d, respectively.

### 3.2. Results of Regression Model

#### 3.2.1. Relationship between the Risk of Viral Diarrhea Infection and the Average Temperature

**Figure 2:**
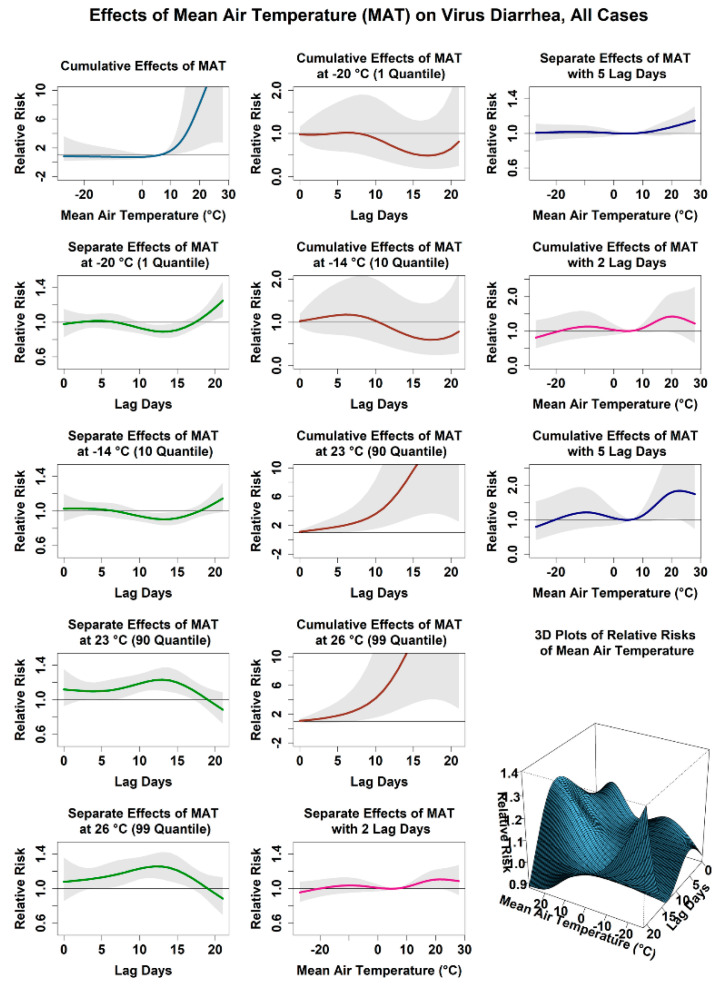
Effect of mean air temperature on virus diarrhea in Jilin Province, all cases.

Figure 2 shows the relationship between the attributed infection risk of viral diarrhea among children under 5 years old and the average temperature. In this study, 6 °C, the mean daily average temperature in Jilin Province, was set as the reference value. We found a complex relationship between the attributed risk of viral diarrhea among children and the average temperature. The cumulative risk at low temperature (−14 °C, 10 quantile) was 0.78 (0.28, 2.15), while that at high temperature (23 °C, 90 quantile) was 11.16 (2.52, 49.45). The cumulative risk reaches the minimum at the temperature of −3 °C (30 quantile). We observed the relative risk and cumulative risk for viral diarrhea infection of different lag days under low (−14 °C) and high temperature (23 °C). Under low temperature, the infection risk of viral diarrhea was 1.02 (0.94, 1.10) on the fifth lag day and decreased to 0.92 (0.85, 1.00) on the 15th lag day. The cumulative risk reached the peak of 1.18 (0.69, 2.00) on the sixth day, began to decline, and decreased to 1.18 (0.69, 2.00) on the 15th day. Under high temperature, the infection risk of viral diarrhea was 1.10 (1.00, 1.21) on the fifth lag day and increased to 1.2 (1.07, 1.34) on the 15th lag day. The cumulative risk increased with the delay time, reaching 13.34 (3.47, 51.3) after 19 days. Furthermore, under the condition of extremely high temperature (26 °C, 99 quantile), the cumulative risk reached its peak of 14.35 (2.78, 74.14), and the infection risk reached its peak of 1.21 (1.06, 1.38) on the 15th lag day. Stratified analysis of different subgroups showed that the average temperature had different exposure risks to varying gender and age groups, as shown in Figures S1–S5 in the Supporting Information. The cumulative risk of low temperature for males was 0.67 (0.18, 2.50), while that for females was 0.92 (0.20, 4.26), and the cumulative risk for females at −20 °C reached 1.32 (0.22, 7.92). For different age groups, the cumulative risk at low temperatures was lower for children under 1 year old but higher for children over 1 year old. Similar to the general situation, the low temperature conditions showed consistent relative risks to different populations. The relative risks of infection of different populations on the fifth lag day were generally higher than those on the 15th lag day. The cumulative effect of low temperature in different populations also showed a phenomenon of first increasing and then decreasing with the extension of lag time. The cumulative risks of high temperatures on different populations increased with the extension of lag time, which was consistent with the overall situation.

#### 3.2.2. Relationship between the Infection Risk of Viral Diarrhea and Precipitation

**Figure 3:**
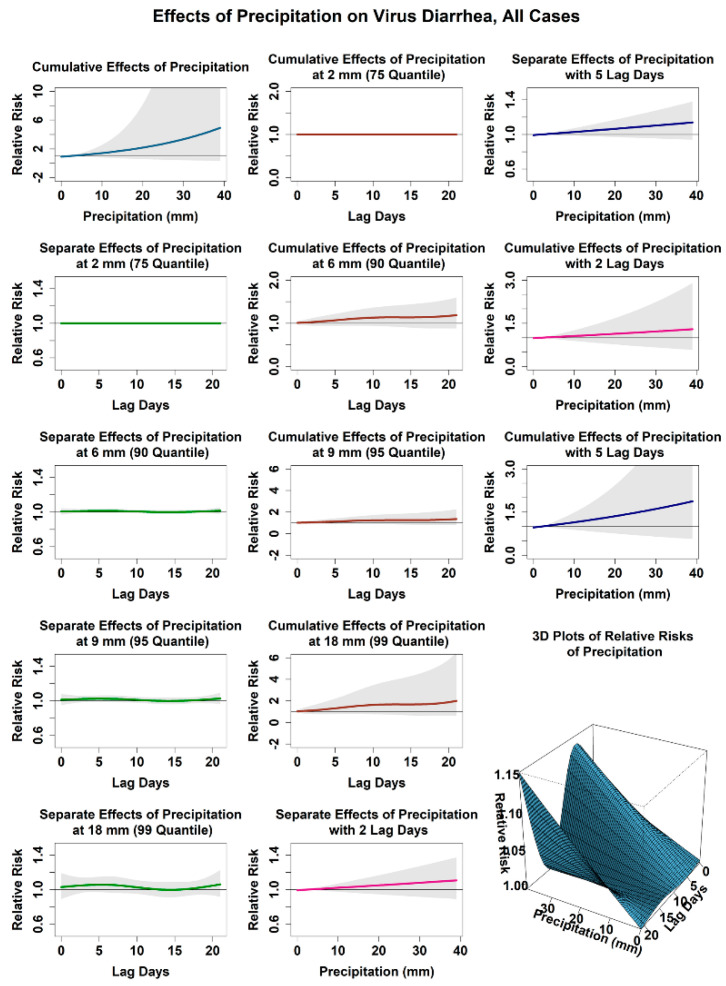
Effect of precipitation on virus diarrhea in Jilin Province, all cases.

Figure 3 shows the relationship between the attributed infection risks of viral diarrhea among children under 5 years old and daily precipitation. In this study, 2 mm, close to the average value of daily precipitation in Jilin Province, was set as the reference value. Compared with the average temperature, the relationship between the infection risk of viral diarrhea among children and daily precipitation was simple. The cumulative relative risk increased steadily with the increase in daily precipitation. The cumulative risk was 1.19 (0.89, 1.60) when the daily precipitation was 6 mm (90 quantile) and 1.99 (0.61, 6.48) when the daily precipitation was 18 mm (99 quantile). We observed the relationship between the infection risk of viral diarrhea and the lag days under conditions of 6 mm and 18 mm precipitation. Under the condition of 6 mm precipitation, the relative risk was 1.01 (0.99, 1.03) on the second and the fifth lag days. The cumulative risk increased with the extension of lag time, reaching 1.14 (0.90, 1.44) on the 15th lag day. Under the condition of 18 mm precipitation, the relative risk reached the peak value of 1.06 (0.97, 1.15) on the fifth lag day and fell to 1.00 (0.91, 1.09) on the 15th lag day. The cumulative risk increased with the extension of lag time, and it climbed to 1.67 (0.65, 4.29) on the 15th lag day. Stratified analysis of different subgroups showed that compared with children under 1 year old, children over 1 year old had a greater relative infection risk of within 1 week when the daily precipitation was 6 and 18 mm, as shown in Figures S1–S5 in the Supporting Information. Compared with the daily precipitation of 6 mm, the short-term infection risks of all sub-age groups under daily precipitation of 18 mm were higher than those under daily precipitation of 6 mm. For different gender groups, the infection risk was similar to the overall situation, and the difference in infection risk between male groups and female groups was minimal.

#### 3.2.3. Relationship between the Infection Risk of Viral Diarrhea and AQI

**Figure 4:**
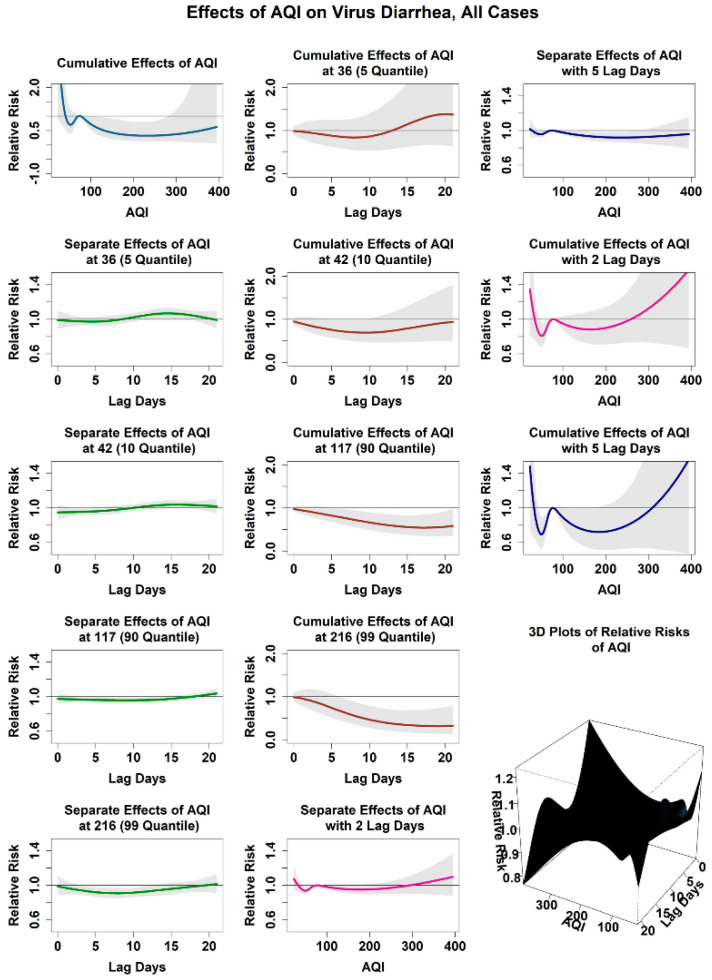
Effect of AQI on virus diarrhea in Jilin Province, all cases.

Figure 4 shows the relationship between the attributed infection risk of viral diarrhea among children under 5 years old and daily AQI. In this study, AQI of 76, close to the mean value of daily AQI in Jilin Province, was set as the reference value. We found a complicated relationship between the infection risk of viral diarrhea among children and AQI. The cumulative infection risk of viral diarrhea among children was 0.58 (0.35, 0.98) when AQI was high (AQI equals to 117, 90 quantile, the same below) and 0.94 (0.49, 1.80) when AQI was low (AQI equals to 42, 10 quantile, the same below). We observed the relationship between the infection risk of viral diarrhea on different lag days among children under AQI of 42 and 117, respectively. When the AQI was low, the relative infection risk was below 1.0 within 1 week. The relative risk was 0.96 (0.92, 1.00) and the cumulative risk was 0.75 (0.57, 1.00) on the fifth lag day. However, the relative risk rose to greater than 1.0 after 10 lag days. The relative risk reached the maximum of 1.04 (0.99, 1.09) on the 15th lag day, while the cumulative risk reached the minimum of 0.69 (0.46, 1.03) on the 10th lag day and then increased slowly thereafter. When the AQI was high, the relative infection risk was 0.96 (0.93, 0.99) on the fifth lag day, but it changed very little within 3 weeks. The cumulative risk decreased gradually with the extension of the lag time, and it dropped to as low as 0.56 (0.37, 0.83) on the 15th lag day. Furthermore, under the condition of extremely high AQI (216, 99 quantile), the cumulative infection risk was reduced to 0.32 (0.13, 0.80). Stratified analysis of different subgroups showed that the infection risks between viral diarrhea among different gender and age groups and AQI were similar to the overall situation, as shown in Figures S1–S5 in the Supporting Information. When AQI was low, the relative risk of infection was generally below 1.0 within 1 week of lag time, but it started to increase after 1 week and reached the peak at about 2 weeks of lag time. Among all the subgroups, the change in the infection risk of males was the smallest. The cumulative risk generally showed a steady downward trend within 1 week of lag time, and it began to increase after about 2 weeks of lag, except in the male groups. When AQI was high, the infection risk of different subgroups generally showed stable characteristics; the relative risks were generally below 1.0, and they changed very little with the extension of lag time. The corresponding cumulative risk exhibited a steady decline. Notably, when AQI was extremely low, the cumulative infection risks of all subgroups greatly increased, and this scenario was similar to the overall situation.

#### 3.2.4. Relationship between the Infection Risk of Viral Diarrhea and the Combined Baidu Index

**Figure 5:**
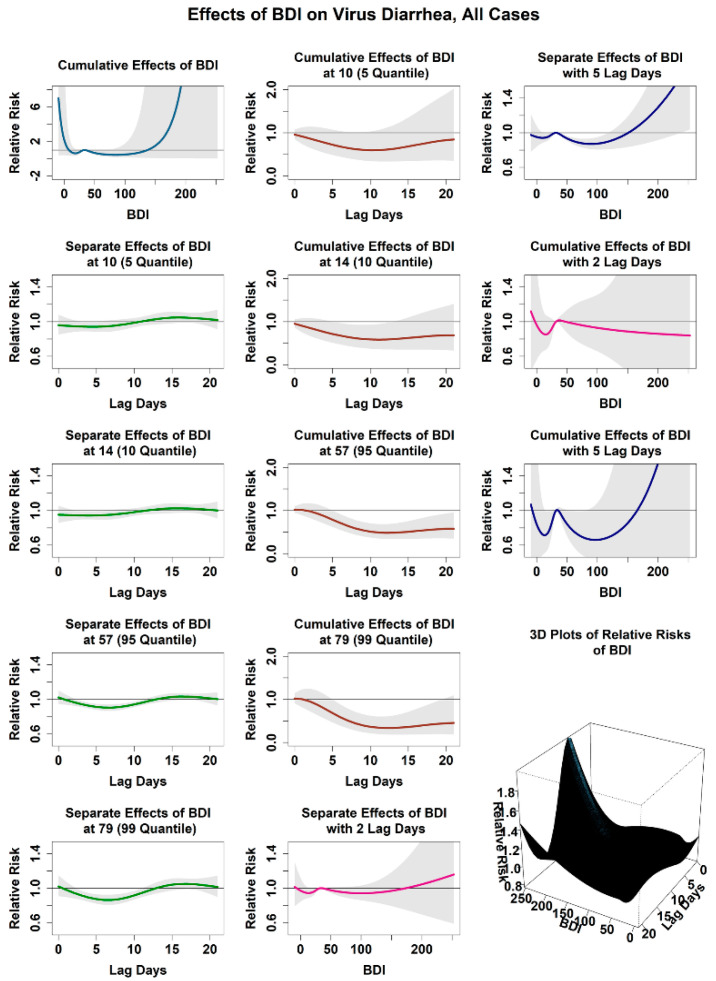
Effect of combined Baidu index on virus diarrhea in Jilin Province, all cases.

Figure 5 shows the relationship between the attributed infection risk of viral diarrhea among children under 5 years old and the combined Baidu index. In this study, the combined BDI of 32, close to the mean value of the combined BDI in Jilin Province, was set as the reference value. The cumulative relative risk was generally lower than 1.0 when the combined BDI ranged from 10 to 57 (between 5 and 95 quantiles). For example, when the combined BDI was 10, the cumulative risk was 0.84 (0.35, 2.02); it reached 0.58 (0.35, 0.96) and 0.45 (0.19, 1.09) when the combined BDI was 57 and 79 (99 quantile), respectively. However, the cumulative risk increased rapidly with a further increase in the combined BDI. We observed the relationship between the infection risk of viral diarrhea among children and the lag days under the combined BDI of 10 and 57. The infection risk was usually lower than 1.0; it reached the minimum of 0.95 (0.89, 1.02) and 0.90 (0.87, 0.94) on the seventh day, respectively, and then gradually rose to the maximum of 1.05 (0.98, 1.12) and 1.03 (0.99, 1.07), respectively. The cumulative risk dropped steadily to about 0.5 within 10 days and then increased slightly. Meanwhile, we found that the cumulative risk reached a very high level when the combined BDI was minimal or near the maximum. For example, the cumulative risk was as high as 12.16 (0.04, 3962.84) when the combined BDI was 200. Stratified analysis of different subgroups showed that the relationship between viral diarrhea and the combined BDI in different populations exhibited similar properties, as shown in Figures S1–S5 in the Supporting Information. The cumulative risk was generally lower than 1.0 when the combined BDI ranged from 5 to 95 quantiles. The relative infection risks were generally lower than 1.0 within 10 days but reached a maximum after 2 weeks, and the corresponding cumulative risk decreased steadily within 10 days and then increased slowly. We also found that the extreme combined BDI brought greater risks to viral diarrhea infection for the overall situation and all subgroups.

## 4. Discussion

According to previous studies, viral diarrhea exhibited a periodicity with a yearly period all over the world, and its outbreak is related to various environmental factors. For example, meteorological factors and air quality factors may directly affect the persistence or activity of viruses and the activities of humans. We believe that temperature and precipitation are meteorological factors that have a great influence on human activities, while other meteorological factors (such as air pressure and humidity) are collinear with or related to temperature and precipitation to a certain extent. AQI is the result of the comprehensive calculation of the concentrations of various air pollution elements such as PM2.5 and SO_2_. Therefore, AQI represents the overall situation of air quality, and it has an important impact on human activities. In addition, the combined BDI can reflect human attention to specific social events and social activities in a certain area. In this study, the daily average temperature, precipitation, AQI, and combined compound BDI were selected as the external factors for model construction, and the relationship between viral diarrhea infection among children under 5 years old and the four selected factors was studied.

Different from previous research, this study covered a variety of viral infectious diarrhea, and the time series of infection numbers were the comprehensive performance of infection of a variety of viruses. Therefore, the relationship between infection risk and exposure to various external factors obtained by this study differed from previous research. However, our findings still further support the existing research results. Our work showed that the infection rate of viral diarrhea reached its peak in winter, and the increase in the risk of viral diarrhea caused by low temperature was consistent with the previous reports that low temperature has an important effect on the increase in rotavirus diarrhea infection. Atchison, C. J. [8] found that the risk of rotavirus infection decreases by 4% if the temperature drops by 1 °C in Western Europe. Celik, C. [9] found that the rate of rotavirus infection in Turkey would increase by 0.523% if the temperature dropped by 1 °C. Laboratory environmental evidence showed that virus particles were more stable at low temperatures [31], which could make them last longer on human hands, feces, and other contaminated objects. In addition, as rotavirus can be atomized [32], the virus can spread through dust suspended in the air. However, the biological reasons underlying the high transmission rate of rotavirus infection at low temperatures remain unclear [33]. People may tend to reduce outdoor activities in cold winters, which increases the frequency of contact. Meanwhile, changes in living habits, such as the decrease in handwashing frequency in cold conditions, may increase the chance of virus transmission through contact. In this study, we found that low temperature could improve the risk of viral diarrhea infection among children within 1 week to approximately 10 days, showing obvious short-term effects. Therefore, in winter, when dealing with viral diarrhea among children, epidemic prevention and control departments and the public should pay special attention to the risk of viral diarrhea outbreak in the short term when the temperature drops.

We found that high temperatures could also promote an infection rate of viral diarrhea among children. We believe this phenomenon results from the fact that the cases of viral diarrhea collected in our work included various kinds of viral infection cases, and summer is usually the season of the high incidence of norovirus. We believe it is related to the climate characteristics of the selected study area in summer. It belongs to the temperate monsoon climate in Jilin Province, and summer is short but hot with heavy rain. Wang, P. [18] found a positive correlation between daily precipitation and diarrhea caused by norovirus, and the correlation was stronger in summer than in other seasons. The risk of hospitalization under the condition of 34.1 mm precipitation was 2.95, which was 1.6 times higher than that in winter. Heavy precipitation can increase the runoff of local rivers. Greer, A. L. [16] found that the increase in river runoff can promote the outbreak of norovirus. Although virus activity decreases with the increase in air temperature, frequent rainfall in summer may make rivers and groundwater sources contact pollutants more easily. High microbial loads are likely found in sewage overflow or damaged drainage systems where pollutants are concentrated, thereby making aquatic products more susceptible to virus pollution and resulting in the outbreak of foodborne virus diarrhea [34]. When untreated polluted water is used for entertainment, children are particularly susceptible to infection, and this association is strongest in summer than in other seasons. We found that the increase in daily precipitation led to a steady increase in the infection risk of viral diarrhea among children under 5 years old, while the increase in temperature on the infection risk may be an indirect factor. We also found that high temperature and precipitation had a long-term effect on viral diarrhea infection risk, and the cumulative risk increased steadily with the increase in lag time. Therefore, in the short summer, the epidemic prevention and control departments and the public in Jilin Province need to do a good job in preventing viral diarrhea among children for a long time.

The influence of AQI on the infection risk of viral diarrhea among children was studied in this paper. To our surprise, we found that the promotion of AQI had a negative effect on the infection risk of viral diarrhea within 1 week to approximately 10 days for the overall situation and all the subgroups. Although the cumulative infection risk increased sharply when AQI was extremely low, no statistical significance was found between AQI and viral diarrhea infection data in the corresponding range of AQI (*p* > 0.05). To date, reports on air pollution factors and the risk of viral diarrhea infection are rare. Ye, Q. [20] once found that the concentrations of pollutants, such as CO, SO_2_, PM10, and NO_2_ in the air, are positively correlated with the incidence of rotavirus, and these pollutants significantly increase the relative risk of rotavirus infection in children, while showing obvious dose, lag, and cumulative effects. Numerous studies have shown that air pollution may be the cause of the high incidence of respiratory diseases [35], but the way in which air pollution affects fecal-transmitted digestive tract diseases through the main transmission routes remains inconclusive. In our work, within 10 lag days, higher AQI inhibited the infection risk of viral diarrhea among children under 5 years old compared with the average daily AQI, and the relative risk did not rise to more than 1.0 until 2 weeks later. The relationship between diarrhea and air pollutants includes direct and indirect mechanisms. The former refers to the fact that the virus can cause intestinal infection. Several studies have shown that rotavirus, norovirus, and other pathogens that cause diarrhea can be transmitted through the air to form aerosols [4], and holes on the surface of the PM10 particles can carry viruses according to the electron microscope photographs [36]. Enterovirus carried by excreta of patients and the external environmental pollutants such as water and soil can spread through inhalable particulate matter, and these particles can contaminate the living environment of children and increase the infection risk of fecal-transmitted digestive tract diseases. The indirect mechanism is that air pollutants can affect the intestinal environment and then harm the immune system, thus increasing the risk of viral infection. For example, exposure to high doses of PM10 can result in the death of intestinal epithelial cells, the increase of intestinal permeability [37], leading to intestinal inflammation, and the ingested air pollutants significantly influence the gut microbe composition and metabolic processes. Gaseous pollutants such as NO_2_ may decrease the specific immunity and may induce an inflammatory response in the digestive tract mucosa and increase the risk of viral infection [38].

We suspect that the influence of AQI on viral diarrhea infection among children mainly depends on the influence of AQI on children’s daily activities. With the continuous development of Chinese society, more people are beginning to realize the harm of air pollution to human health. When AQI is high, the air quality is poor. China has established an air quality early warning system. When meteorological factors such as high concentration of air pollutants and poor diffusion conditions occur, local air quality monitoring departments will issue air pollution warnings. At this time, adults tend to wear masks to protect themselves and prevent airborne diseases such as the novel coronavirus, which broke out in early 2020 [39], and children are usually arranged by their parents to reduce outdoor activities as much as possible to reduce the probability of transmission of various viruses through pollutants, including inhalable particles carrying the virus, toxic gases that may damage the immune system, and water that may be contaminated by air pollutants. Therefore, the risk of children infected with viral diarrhea is lower within 10 lag days under the condition of high AQI. Although our results may not be completely consistent with the findings of Ye, Q. [20], we believe that this may be related to the change in living habits of the public when the concentration of air pollutants is high in recent years. Even though viruses related to viral diarrhea can be transmitted through air pollutants such as PM10, in recent years, the local public’s emphasis on their health and parents’ emphasis on children’s health have been increasing with the publicity of public health. When the concentration of air pollutants is high, protective tools such as masks are popularized, and the public tends to reduce the frequency of outdoor activities. Therefore, the infection risk of viral diarrhea will drop for several days under high AQI. In addition, we found that the relative risk began to increase after two lag weeks, which may be caused by the public’s efforts to prevent and control airborne diseases beginning to decrease after the end of the air pollution period. Therefore, when air pollution is serious, we believe that the public’s own prevention and control measures can inhibit the outbreak of viral diarrhea among children; however, after a period of pollution, the public must pay attention to the re-infection of viral diarrhea.

In view of the analysis of network search and viral diarrhea infection, although there have been related studies, the existing reports are usually limited to relatively simple data fitting such as correlation analysis [25]. Research using the dlnm model has not been reported yet. In our work, based on the existing research, composite network search data were incorporated into the dlnm model to determine the lag effect of network search data on epidemics. We found that only extreme network search conditions had a positive correlation with infection risk of viral diarrhea among children under 5 years old. We believe that the network search data reflect the public’s concern about social events. When the search index related to a certain disease increases sharply, it may be the concentrated outbreak period of this kind of disease. Therefore, network search data can be used as the key element of early warning of disease outbreaks. In our work, when the complex BDI of viral diarrhea exceeded 150, the relative risk and cumulative risk increased rapidly, meaning that viral diarrhea may have occurred in corresponding areas at this time. As the outbreak period of viral diarrhea usually lasts for several days or more, the sudden increase in search index can help the disease prevention and control department make high-risk early warnings in time and provide instructions for the catering and other industries to quickly monitor foodborne viruses.

The adoption of viral diarrhea data from just one province may be a limitation of our research. As the location information of patients in the original data could not be further concretized, we averaged the external factors monitored by several monitoring points in Jilin Province. Therefore, our work can only reflect the relationship between the infection risk of viral diarrhea and external factors in a relatively large area, but it does not make the research area accurate to a city or a smaller area. In addition, to avoid the complexity of our model, we selected the external factors that may have a greater impact on public activities, including daily average temperature, precipitation, AQI, and daily combined BDI, among which the first three were natural environmental factors and the last one was a social factor. Other external factors, such as other natural environmental factors, including meteorological factors and specific air pollutants that may have an impact on public activities, and other sociological factors, including economy, nutritional condition, and population mobility, have not been included in our model. Therefore, more targeted research will be conducted in the future to show the relationship between children’s viral diarrhea and external factors in more detail.

## 5. Conclusions

Our research further proved an intense relationship between viral diarrhea infection and temperature, precipitation, air quality, and social concern in temperate zones. In Jilin Province, China, low temperature has a short-term effect on the increase in viral diarrhea infection risk among children under 5 years old for about 1 week, while high temperature and heavy precipitation have a long-term effect on the increase of infection. In the short term, the increase in AQI may change the living activities of the public and inhibit the occurrence of children’s viral diarrhea, but the risk of infection will increase after 2 weeks. To cope with the outbreak of viral diarrhea among children, disease prevention and control departments and the public need to pay close attention to natural environmental factors. The occurrence of extreme network search index can reflect the local outbreak of viral diarrhea, which can help the epidemic prevention and control department release early warnings of high risk of an outbreak in time.

## Figures and Tables

**Table 1:** Descriptive statistics of daily number of infection cases of each group, and the selected four external factors.

Title 1	Mean	Std	Min	5%	25%	50%	75%	95%	Max
All	1.28	1.39	0	0	0	1	2	4	10
Male	0.74	1	0	0	0	0	1	3	8
Female	0.53	0.8	0	0	0	0	1	2	5
0–1 Years Old	0.53	0.79	0	0	0	0	1	2	5
1–2 Years Old	0.49	0.79	0	0	0	0	1	2	4
2–5 Years Old	0.25	0.56	0	0	0	0	0	1	5
Mean Air Temperature (°C)	6.05	13.76	−27.08	−16.29	−7.11	8.13	18.63	24.09	28.16
Precipitation (mm)	1.75	3.88	0	0	0	0.11	1.59	8.98	39.11
Air Quality Index	75.72	38.08	22.44	36.11	52.33	66.56	87.94	147.28	394.5
Combined Baidu Index	32.49	16.92	−9.62	10.32	21.47	30.8	41.6	57.34	252.55

## Data Availability

The National Institute for Viral Disease Control and Prevention of China has data on children’s viral diarrhea cases in Jilin Province, China. Due to the agreement, the author cannot disclose data on viral diarrhea cases.

## Supplementary Materials

The following are available online at https://www.mdpi.com/article/10.3390/ijerph182111615/s1, Table S1: The selected 20 keywords about viral diarrhea for Baidu search data acquisition, Figure S1: Effect of 4 Major Factors on Virus Diarrhea, Male, Figure S2: Effect of 4 Major Factors on Virus Diarrhea, Female, Figure S3: Effect of 4 Major Factors on Virus Diarrhea, 0–1 Years Old, Figure S4: Effect of 4 Major Factors on Virus Diarrhea, 1–2 Years Old, Figure S5: Effect of 4 Major Factors on Virus Diarrhea, 2–5 Years Old.
