# Supplementary material for: Short-Term Impacts of Meteorology, Air Pollution, and Internet Search Data on Viral Diarrhea Infection among Children in Jilin Province, China
Source: Int J Environ Res Public Health. 2021 Nov 4;18(21):11615. doi: 10.3390/ijerph182111615 (PMC8582928; doi:10.3390/ijerph182111615)

Supporting Information

Table S1: The selected 20 keywords about viral diarrhea for Baidu search data acquisition

Title (Chinese)	Interpretation in English	Title (Chinese)	Interpretation in English
84 消毒液	84 Disinfectant	急性胃肠炎	Acute Gastroenteritis
口服补液盐	Salesre Hydrationi Soralis	益生菌	Probiotics
合生元	Synbiotics	腺病毒	Adenovirus
妈咪爱	Medilac-Vita	蒙脱石散	Montmorillonite Powder
尿不湿	Diapers	诺如病毒	Norovirus
消毒液	Disinfectant	轮状病毒	Rotavirus
湿巾	Wet Wipes	腹泻	Diarrhea
滴露	Dettol	腹痛	Stomachache
生蚝	Oyster	呕吐	Vomiting
牡蛎	Oyster	发热	Fever

Figure S1: Effect of 4 Major Factors on Virus Diarrhea, Male

Effects of Mean Air Temperature (MAT) on Virus Diarrhea, Male

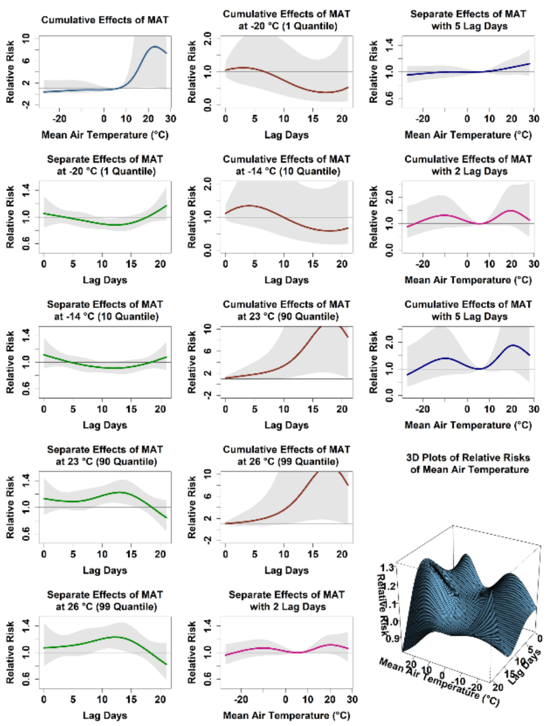

Effects of Precipitation on Virus Diarrhea, Male

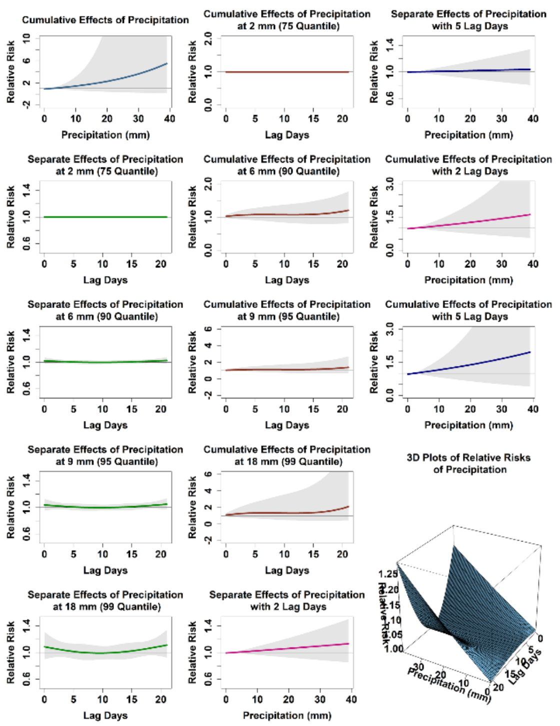

Effects of AQI on Virus Diarrhea, Male

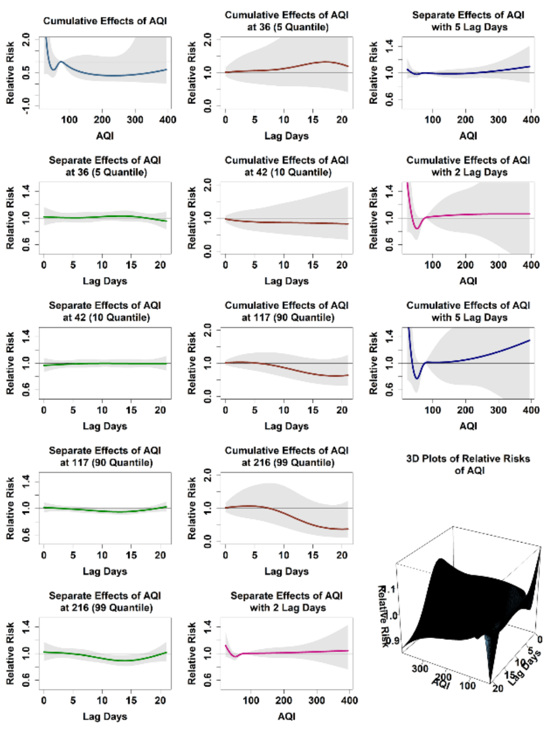

Effects of BDI on Virus Diarrhea, Male

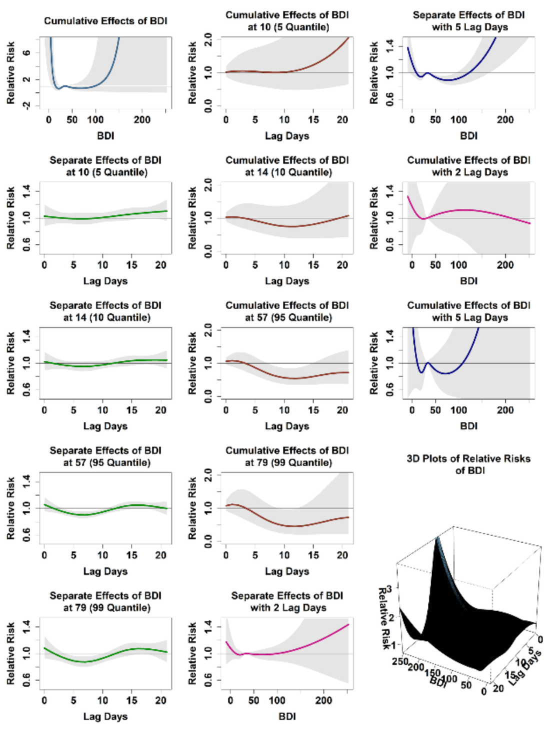

Figure S2: Effect of 4 Major Factors on Virus Diarrhea, Female

Effects of Mean Air Temperature (MAT) on Virus Diarrhea, Female

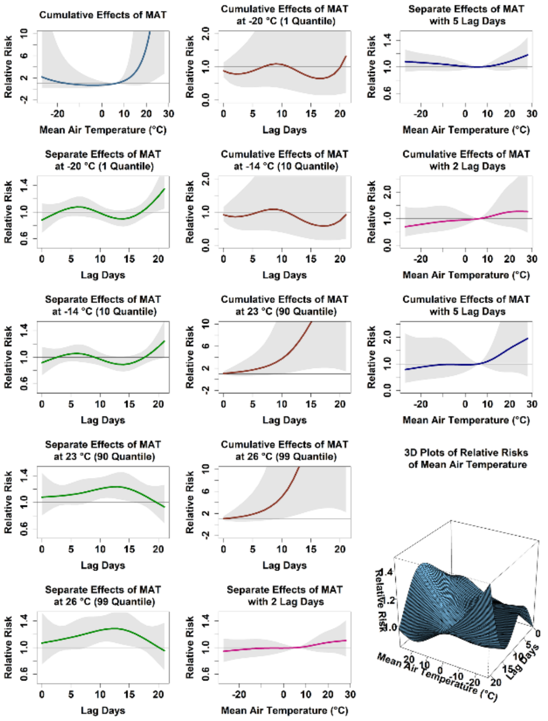

Effects of Precipitation on Virus Diarrhea, Female

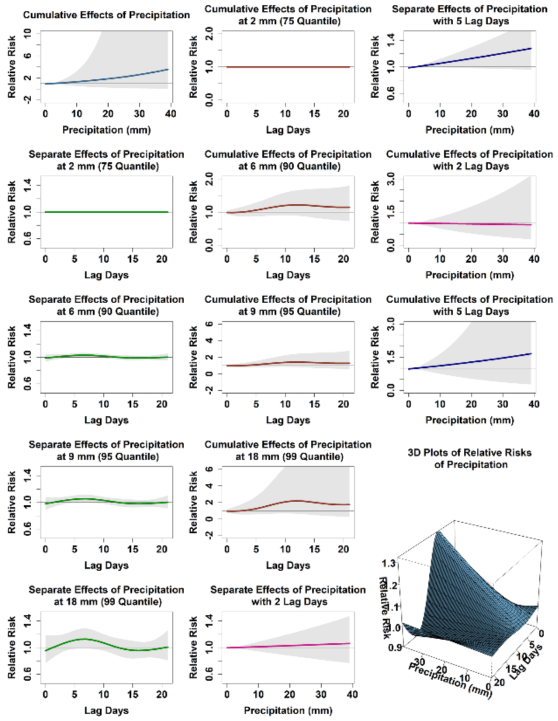

Effects of AQI on Virus Diarrhea, Female

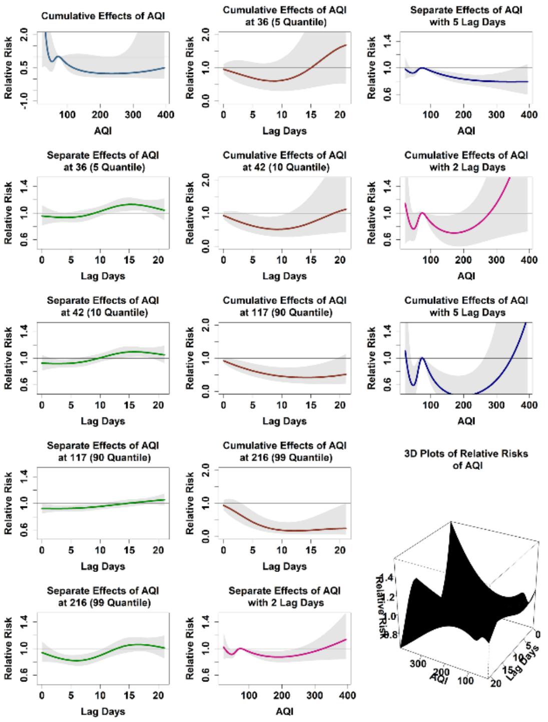

Effects of BDI on Virus Diarrhea, Female

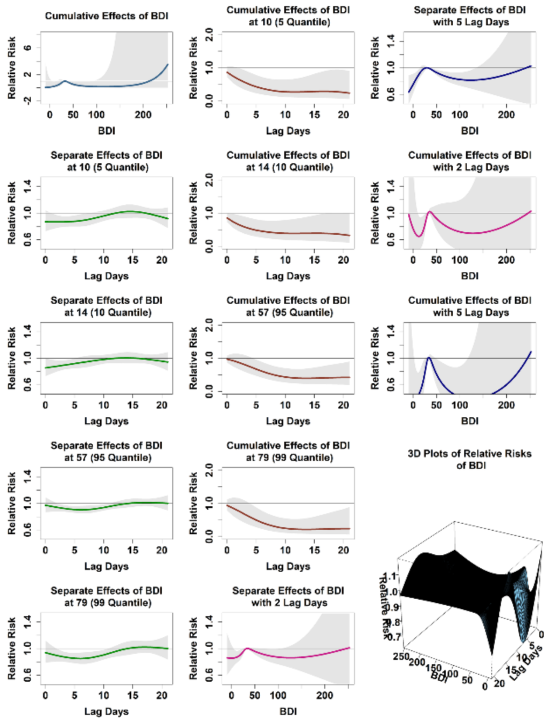

Figure S3: Effect of 4 Major Factors on Virus Diarrhea, 0-1 Years Old

Effects of Mean Air Temperature (MAT) on Virus Diarrhea, 0-1 Years Old

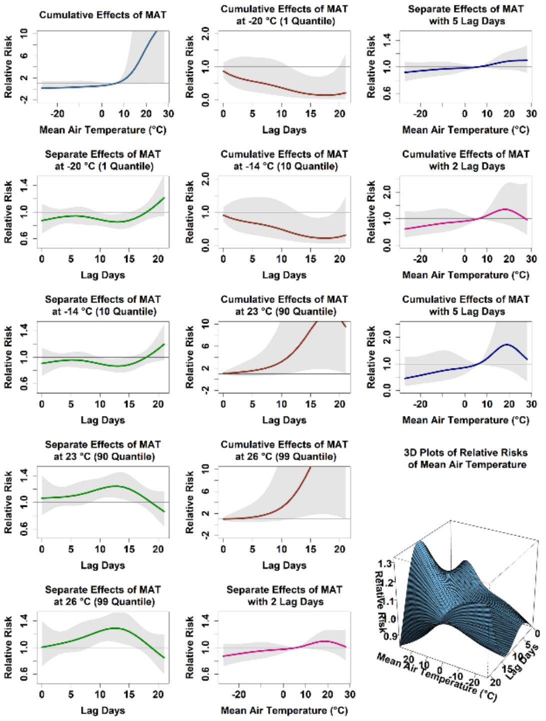

Effects of Precipitation on Virus Diarrhea, 0-1 Years Old

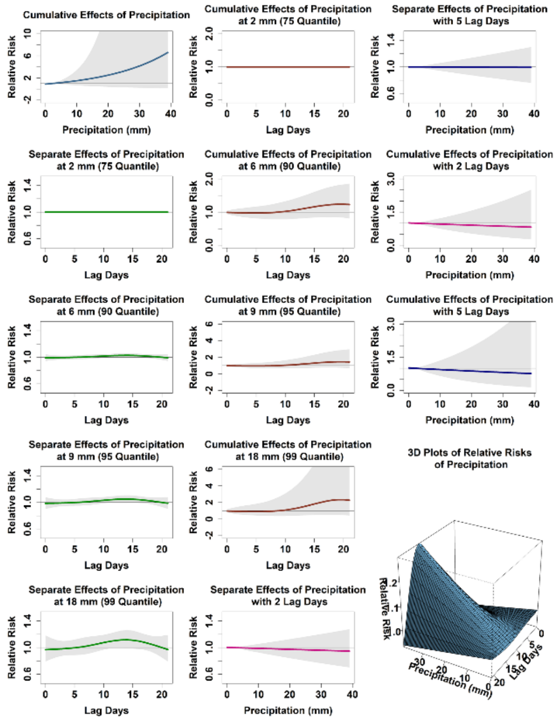

Effects of AQI on Virus Diarrhea, 0-1 Years Old

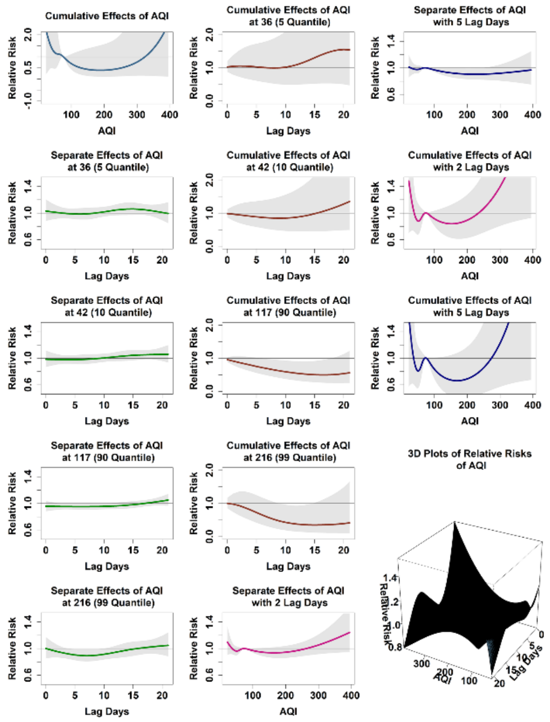

Effects of BDI on Virus Diarrhea, 0-1 Years Old

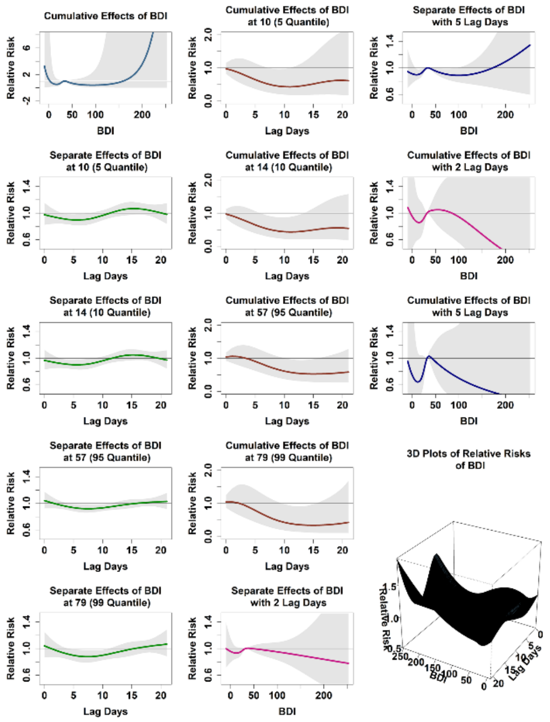

Figure S4: Effect of 4 Major Factors on Virus Diarrhea, 1-2 Years Old

Effects of Mean Air Temperature (MAT) on Virus Diarrhea, 1-2 Years Old

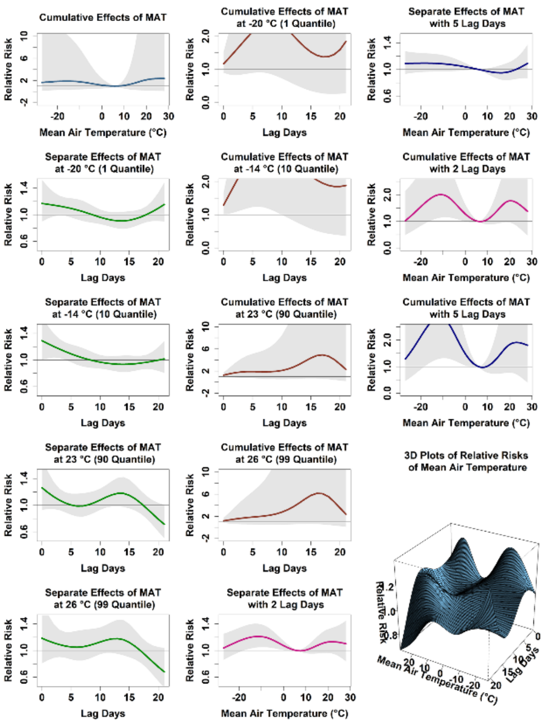

Effects of Precipitation on Virus Diarrhea, 1-2 Years Old

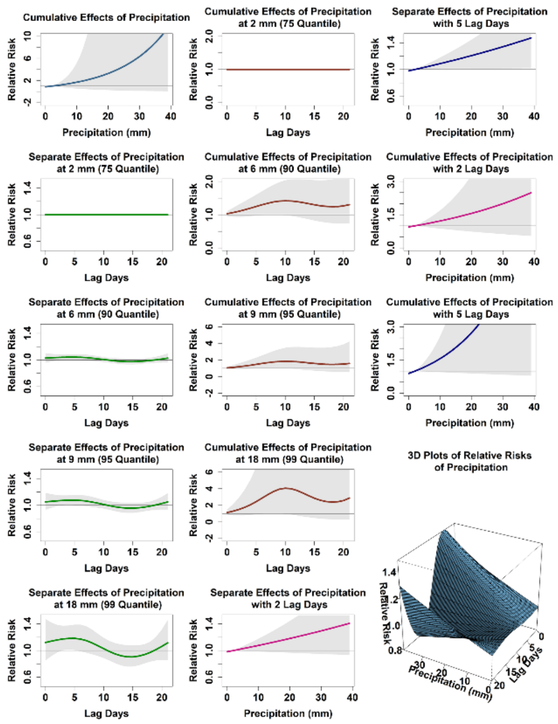

Effects of AQI on Virus Diarrhea, 1-2 Years Old

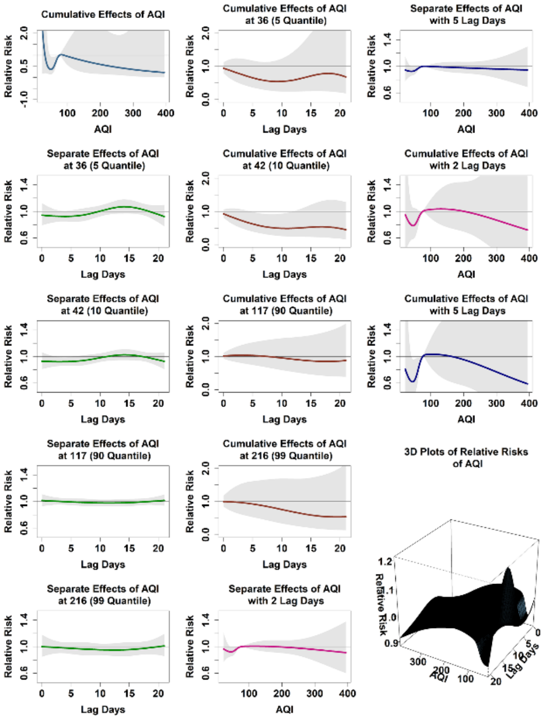

Effects of BDI on Virus Diarrhea, 1-2 Years Old

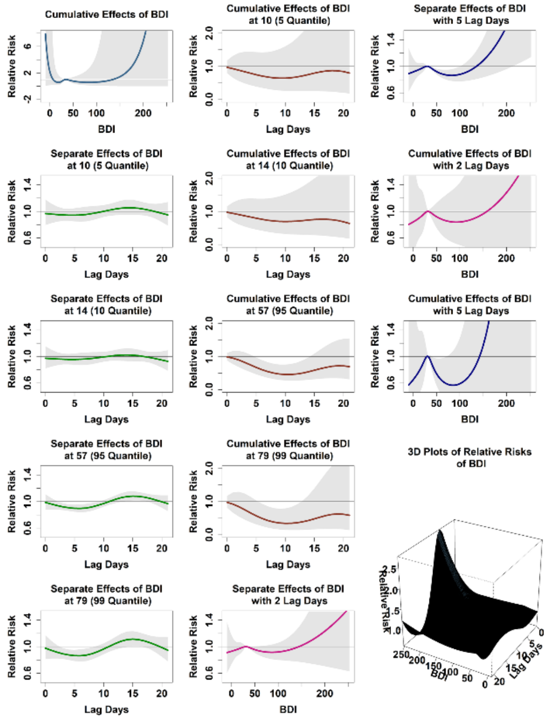

Figure S5: Effect of 4 Major Factors on Virus Diarrhea, 2-5 Years Old

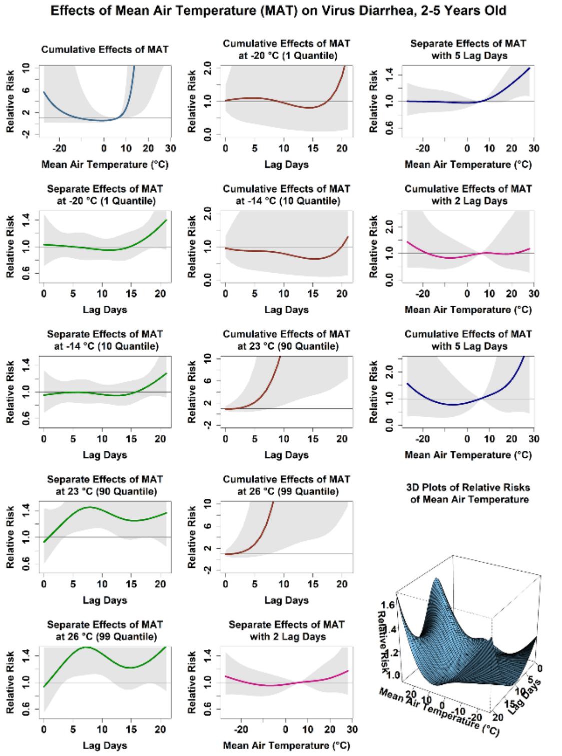

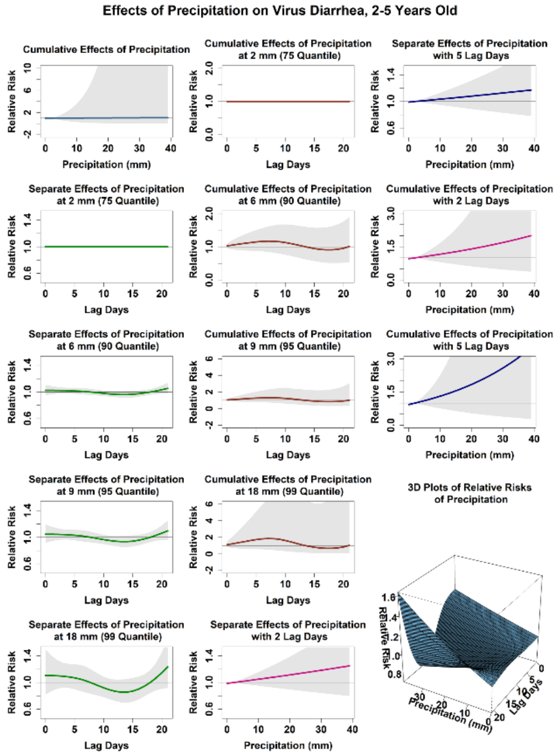

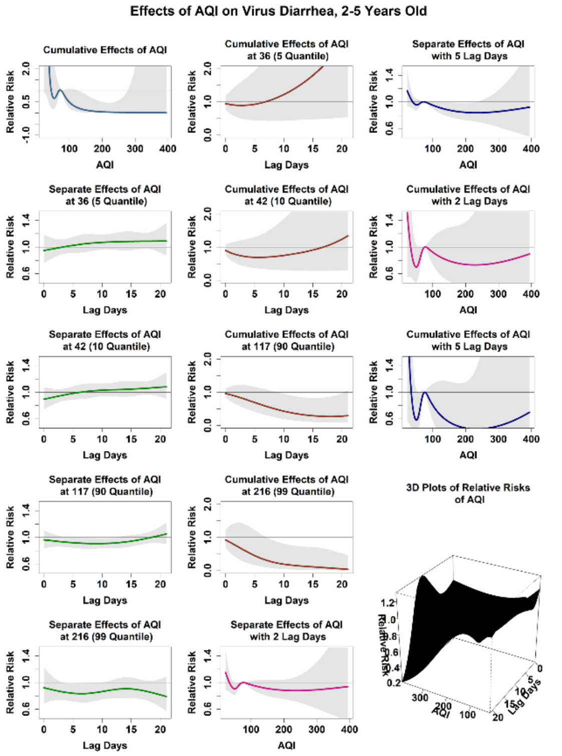

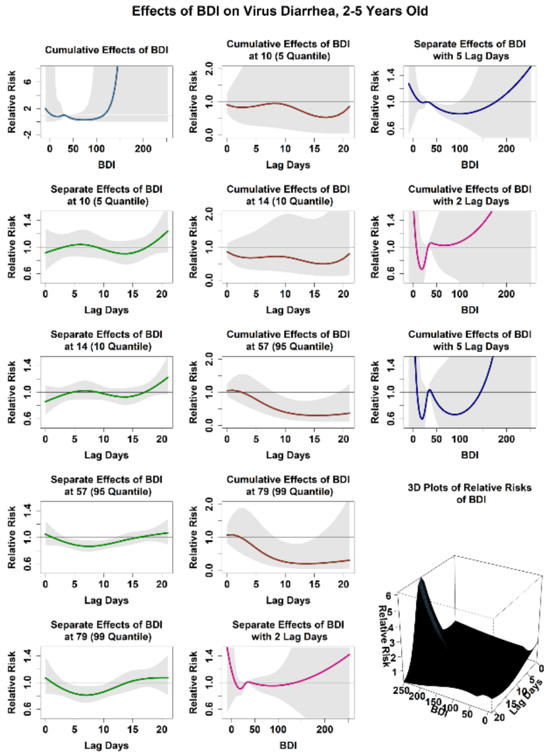

Supplement: Supplementary file 1 [file ijerph-18-11615-s001.zip › ijerph-1379342-supplementary.pdf]
